# Cucumber grafting on indigenous cucurbit landraces confers salt tolerance and improves fruit yield by enhancing morpho-physio-biochemical and ionic attributes

**DOI:** 10.1038/s41598-023-48947-z

**Published:** 2023-12-07

**Authors:** Fazal Abbas, Hafiz Nazar Faried, Gulzar Akhtar, Sami Ullah, Talha Javed, Muhammad Asif Shehzad, Khurram Ziaf, Kashif Razzaq, Muhammad Amin, Fahad Masoud Wattoo, Aqsa Hafeez, Mehdi Rahimi, Amany H. A. Abeed

**Affiliations:** 1 Department of Horticulture, MNS University of Agriculture, Multan, Pakistan; 2 College of Agriculture, Fujian Agriculture and Forestry University, Fuzhou, 350002 China; 3 Institute of Plant Breeding and Biotechnology, MNS University of Agriculture, Multan, Pakistan; 4 Institute of Horticultural Sciences, University of Agriculture, Faisalabad, Pakistan; 5 Department of Horticultural Sciences, The Islamia University of Bahawalpur, Bahawalpur, Pakistan; 6 Department Plant Breeding and Genetics, PMAS Arid Agriculture University, Rawalpindi, Pakistan; 7 Department of Plant Sciences, Quaid-i-Azam University, Islamabad, 45320 Pakistan; 8 Department of Biotechnology, Institute of Science and High Technology and Environmental Sciences, Graduate University of Advanced Technology, Kerman, Iran; 9 Department of Botany and Microbiology, Faculty of Science, Assiut University, Assiut, 71516 Egypt

**Keywords:** Ecology, Plant sciences

## Abstract

Pakistan is the 8th most climate-affected country in the globe along with a semi-arid to arid climate, thereby the crops require higher irrigation from underground water. Moreover,  ~ 70% of pumped groundwater in irrigated agriculture is brackish and a major cause of secondary salinization. Cucumber (*Cucumis sativus* L.) is an important vegetable crop with an annual growth rate of about 3.3% in Pakistan. However, it is a relatively salt-sensitive crop. Therefore, a dire need for an alternate environment-friendly technology like grafting for managing salinity stress in cucumber by utilizing the indigenous cucurbit landraces. In this regard, a non-perforated pot-based study was carried out in a lath house to explore indigenous cucurbit landraces; bottle gourd (*Lagenaria siceraria*) (cv. Faisalabad Round), pumpkin (*Cucurbit pepo.* L) (cv. Local Desi Special), sponge gourd (*Luffa aegyptiaca*) (cv. Local) and ridge gourd (*Luffa acutangula*) (cv. Desi Special) as rootstocks for inducing salinity tolerance in cucumber (cv. Yahla F1). Four different salts (NaCl) treatments; T_0_ Control (2.4 dSm^–1^), T_1_ (4 dSm^–1^), T_2_ (6 dSm^–1^) and T_3_ (8 dSm^–1^) were applied. The grafted cucumber plants were transplanted into the already-induced salinity pots (12-inch). Different morpho-physio-biochemical, antioxidants, ionic, and yield attributes were recorded. The results illustrate that increasing salinity negatively affected the growing cucumber plants. However, grafted cucumber plants showed higher salt tolerance relative to non-grafted ones. Indigenous bottle gourd landrace (cv. Faisalabad Round) exhibited higher salt tolerance compared to non-grafted cucumber plants due to higher up-regulation of morpho-physio-biochemical, ionic, and yield attributes that was also confirmed by principal component analysis (PCA). Shoot and root biomass, chlorophylls contents (a and b), activities of superoxide dismutase (SOD), catalase (CAT) and peroxidase (POX) enzymes, antioxidants scavenging activity (ASA), ionic (↑ K and Ca, ↓ Na), and yield-related attributes were found maximum in cucumber plants grafted onto indigenous bottle gourd landrace. Hence, the indigenous bottle gourd landrace ‘cv. Faisalabad round’ may be utilized as a rootstock for cucumber under a mild pot-based saline environment. However, indigenous bottle gourd landrace ‘cv. Faisalabad round’ may further be evaluated as rootstocks in moderate saline field conditions for possible developing hybrid rootstock and, subsequently, sustainable cucumber production.

## Introduction

Cucumber (*Cucumis sativus* L.) is an economically important vegetable crop. It is enriched with essential nutrients, vitamins, minerals, and bioactive compounds^1^. Globally, the production of cucumber is about 100 million tons from an area of about 3.7 million hectares^2^. In Pakistan, it is cultivated on an area of about 4.6 thousand hectares and has an estimated annual production of around 61 thousand tonnes with an annual growth rate of 3.3%^3^. Three cucumber crops {1. Winter-spring (November- April), 2. Summer (March/April-July), and 3. Autumn crops (August -November)} are grown under protected cultivation systems in Pakistan's arid to semi-arid climate. Thereby, the crops require higher irrigation from underground water. However, ~ 70% of pumped groundwater in irrigated agriculture is brackish and a major cause of secondary salinization^4^ along with an annual increment of about 40,000 ha. Besides, Pakistan is the 8th most climate-affected country in the globe^5,6^. Hence, the cucumber crop has been facing several soil-based biotic and abiotic challenges including salinity^7^.

Salinity leads to soil toxicity problems. Na^+^, Ca^2+^, and Mg^2+^ (Cations) and Cl^–^, SO_4_^2–^, and HCO^3–^ (anions) are the major salinity-causing ions. Na^+^ and Cl^–^ are the most abundant and deleterious ions with maximum solubility^7^. About 320, 80, and 50 million ha of land are affected by salinity in Asia, Africa, and Europe, respectively. About 6.30 million ha of irrigated cultivated lands are salt-affected in Pakistan^8,9^. Salinity reduces up to 50% of agricultural productivity^10,11^, particularly vegetable crops^12^. Cucumber is a relatively salt-sensitive crop with a threshold level of 2.5 dSm^–1^. An increase of each electrical conductivity (EC) unit above the threshold (2.5 dSm^–1^)^13^ causes about 13% reduction in cucumber growth and productivity^14^. High salt concentration in growth medium negatively affects the plant biomass, leaf area, net photosynthetic rate, water use efficiency, phosphorus, and potassium contents, alters metabolic activities, and osmotic functions in cucumber^15,16^. Additionally, higher salinity stress causes physiological drought^17^, disrupts cell ionic balance, and hinders protein synthesis^18^. Sodium (Na^+^) movement (efflux and influx), uptake and compartmentation in plant cells and tissues involve complex transporters (e.g. HKT1 & HKT2) and channels’ network^19^. Therefore, it is necessary to cope with salt stress to attain appropriate cucumber crop growth and productivity.

Salt stress can be managed by adopting different environment-friendly strategies such as genetic, agronomic fortification, and grafting onto relatively tolerant rootstocks^20^. Grafting (an eco-friendly surgical horticulture technique) of cucumber on suitable rootstock improves the crop growth and productivity by increasing the morphological (root tip, diameters, and length), physiological (photosynthetic rate, water use efficiency) and biochemical (SOD, CAT and POX) indices^21,22^. Grafting helps crop plants to tolerate salt stress by replacing the sensitive crop roots with tolerant genotypes species, and decreasing the production losses^23^ via reducing the Na^+^ and enhancing the K^+^ uptake through the leaves and stems. Thus, maintains appropriate K^+^: Na^+^. Pumpkin and bottle gourd exhibit higher salt tolerance by reducing the Na^+^ transport from root to shoot than that of melon and cucumber^24^. Grafting sustains cucumber productivity by utilizing an appropriate rootstock. Generally, various cucurbit rootstock species respond differently to salt tolerance^25^. However, the salt tolerance induction through grafting in many crops, including cucumber^22^, melon, and watermelon^26^, mainly focused on utilizing one cucurbit rootstock for one crop^22^. Therefore, an elaborative work to evaluate the potential indigenous cucurbit landraces as rootstocks (sponge gourd, ridge gourd, pumpkin, bottle gourd) under various salinity levels for cucumber salt tolerance induction is essential.

Although, different reports have described the positive responses of grafting on cucumber growth, physiology, and productivity by utilizing the various cucurbit rootstocks; however, the grafting impact of indigenous cucurbit landraces (e.g. bottle gourd, pumpkin, sponge, and ridge gourd) as rootstocks under various saline growing environments is limited explored. Further, indigenous cucurbit landraces used in the present study have evolved independently through selection without exotic genetic mixing followed by adaptability and acclimatization. These landraces are best adapted to the arid to semi-arid climate of Pakistan (i.e. bottle gourd (cv. Faisalabad Round), pumpkin (cv. Local Desi Special), sponge gourd (cv. Local), and ridge gourd (cv. Local Desi Special) grow vigorously throughout the year while cucumber only during an appropriate growing environment. Hence, we hypothesized that cucumber grafting onto indigenous cucurbit landraces as rootstocks may induce salt tolerance in cucumber. Specifically, the current study was carried out to screen out the indigenous cucurbit landraces as rootstocks and to further assess the potential of grafted cucumber plants for their improved morphological, physio-biochemical, ionic, and yield indices under various salinity environments.

## Materials and methods

### Experimental site and planting materials

The study was conducted in a plant propagation and physiology lab (nursery development, grafting, and healing) and the lath house (pot culture), MNS University of Agriculture, Multan (MNS-UAM) (latitude 31° 8′ 26.93" N and longitude 71°26′ 35.43" E) Pakistan using completely randomized design (CRD) with a two-factor factorial arrangement having four treatments; T0 Control (2.4 dSm^–1^), T1 (4 dSm^–1^), T2 (6 dSm^–1^) and T3 (8 dSm^–1^) with four replications. In this experiment, indigenous cucurbit landraces; sponge gourd (*Luffa aegyptiaca*) (cv. Chikni), bottle gourd (*Lagenaria siceraria*) (cv. *Faisalabad Round*), pumpkin (*Cucurbit pepo*. L) (cv. Desi Special) and ridge gourd (*Luffa acutangula*) (cv. Desi Special) were explored as rootstocks for salt tolerance induction in cucumber (cv. Yalla F1). Seeds were obtained from a local seed dealer of Green Gold, Pakistan Pvt. Ltd.

### Grafting protocol and stress treatments

The seeds of indigenous cucurbit landraces (sponge gourd (*Luffa aegyptiaca*) (cv. Chikni), bottle gourd (*Lagenaria siceraria*) (cv. Faisalabad Round), pumpkin (*Cucurbit pepo*. L) (cv. Desi Special) and ridge gourd (*Luffa acutangula*) (cv. Desi Special) were sown one week earlier than that of cucumber (scion) in 128 celled plug trays containing growing media (peat moss). The trays were kept in indoor growth chamber (180 × 180 cm) for healthy and vigorous seedling development. Grafting was carried out after 25 days of seed sowing. Grafted plants were shifted to a partially environment-controlled (21–23 °C temperature) healing chamber (180 × 360 cm) for a period of eight days. 90–95%, 80–85%, 75–80% relative humidity were maintained during 1-4th, 5-6th and 7-8th days, respectively. Partial light was provided during 5-8th days followed by shifting to lath house for a pot-based salinity experiment. Soil, silt, and farmyard manure in equal ratio (1:1:1) were used as growing medium for 12-inches pots. Different salinity levels were developed in pots as per the protocol of Sandoval^27^ before the transplanting of the grafted plants. The grafted cucumber plants were grown in saline environment and fertigated with NPK fertilizer (18:18:18) (VALAGRO Zona Industrial-66041 Chieti, Italy) by mixing 10-g fertilizer in 10 L of water. The plants of 12-inch pots were harvested after 40 days to measure various morpho-physio-biochemical and ionic parameters while 55 days for yield attributes.

### Parameters measurements

#### Biomass attributes

The root (RL) and shoot lengths (SL) and internodal distance (ID) were calculated with measuring tape. Shoot (SFW) and root fresh weights (RFW) were measured with a digital weighing balance (OHAUS Corporation, Parsippany, NJ USA). Number of leaves (NL) was counted manually. Rootstock (RG) and scion girths (SG) were measured by digital Vernier calipers (CE-7400S, Cambridge). Plant shoots and roots sample were collected and dried in dry oven at 70 ℃ for two days to record the shoot (SDW) and root dry weights (RDW) with digital weighing balance (OHASU corporation, Parsippany, NJ, USA).

#### Gaseous exchange parameters

The gaseous exchange attributes; photosynthesis rate (*A*) (µmol CO_2_ m^–2^ s^–1^), stomatal conductance (*gs*) (µmol CO_2_ m^–2^ s^–1^), sub-stomatal CO_2_ (*Ci*) (µmol H_2_O mol^–11^) and water use efficiency (WUE) (mmol CO_2_ mol^–1^ H_2_O) were measured from 3-4th leave of intact growing plant with a Portable Photosynthetic System (CIRAS-3, SW Version 2.00 Console Serial Number: C3F0255 via PP System, Amesbury, MA, USA) from 11:00 am to 3:00 pm. It was operated at ambient leaf temperature of 34.5 °C, photosynthetic photon-flux density at 760 µmol m^–2^ s^–1^, 95 kPa atmospheric pressure, 98 mL.min^–1^ air flow, and 320 µmol. mol^–1^ CO_2_ concentration.

#### Biochemical attributes

The antioxidant scavenging activity (ASA) was measured by adopting the method of Mimica-Dukić et al.^28^. 1 g cucumber leaves were homogenized, added 2 mL of phosphate buffer (pH 7.0) and centrifuged at 9000 rpm for 5 min at 4 °C. About 50µL supernatant and 5 mL DPPH incubated for 30 min at room temperature and added 200 µL in microplates followed by measuring ASA by noting the absorbance at 517 nm on ELIZA reader (Epoch Eliza reader, Winooski, USA). Similarly, superoxide dismutase (SOD) was measured by following the protocol of Štajner & Popović^29^. About 500 µL phosphate buffer (pH 5.0), 200 µL Titron X, 200 µL methionine, 100 µL NBT and 800 µL distilled water dissolved in the test tubes along-with an addition of 100 µL supernatant. The tubes were placed in the laminar airflow hood under UV light for 15 min and added 100 µL riboflavin. 200 µL from this mixture was added in the microplates and placed in ELIZA reader for measuring SOD at 560 nm absorbance. Moreover, total phenolic contents (TPC), catalase (CAT) and peroxidase (POX) were measured by the protocol of Razzaq et al.^30^. Catalase (CAT) was calculated by using the reaction mixture containing enzyme extract (100 µL) and H_2_O_2_ (100 µL) at an absorbance of 240 nm in ELIZA reader. Similarly, POX was determined by adding the 500 µL phosphate buffer (pH 5), 40 mM H_2_O_2_, 20 mM guaiacol and 100µL supernatant followed by noting the absorbance at 470 nm in ELIZA reader. Total phenolic contents (TPC) were determined by mixing of 100 µL supernatant, 200 µL FC reagents, and 800 µL Na2CO3. 200 µL of this mixture was added in micro-plates and absorbance was noted at 765 nm on ELIZA reader. Similarly, chlorophyll (a and b) were measured by a protocol devised by Lichtenthaler et al.^31^. Frozen leave sample (1 g) was homogenized with the help of pistil and mortal and added 5 mL extraction mixture followed by filling the microplates (200 µL) to run on ELIZA Reader at an absorbance of 470, 645 and 662 nm wavelengths. Following formulas were used for the calculation:

$${\text{Chlorophyll a}} = {11}.{\text{24A662}} - {2}.0{\text{4A645}},$$

$${\text{Chlorophyll b}} = {2}0.{\text{13A645}} - {4}.{\text{19A662}}.$$

### Estimation of minerals ions

Sodium (Na^+^), calcium (Ca^2+^) and potassium (K^+^) were determined as per the protocol invented by Estefan et al.^32^. Cucumber leaves and roots’ samples were dried at 110 ℃ for 2 h followed by digestion in nitric acid (HNO_3_) and perchloric acid (HClO_4_). 1 g dried leave and root samples were added in a mixture containing 6 mL HNO_3_ and 3 mL HClO_4_ and kept overnight. After that, samples were heated on hot plate at 165 ℃ for 10 min. Distilled water was added in each volumetric flask to maintained 50 ml volume followed by filtration in Whatman No. 40 filter paper to attain filtrate aliquot. Na^+^, K^+^ and Ca^2+^ (mg kg^–1^) were computed by running the samples in flame photometer (BWB spectrum technologies, UK).

### Yield parameters

Cucumber average fruit length (cm) was calculated with measuring tape. Average fruit diameter (mm) was noted with digital Vernier calipers (Mitutoyo Cor-proration, Kanagawa, Japan). Average fruit weight (g) and yield per plant (g) were measured with the help of digital weighing balance (OHASU corporation, Parsippany, NJ, USA).

### Statistical method

Statistically, the data was analyzed by performing Fischer’s analysis of variance (ANOVA) by using statistical software DASTAT (Version 1.021, Perugia, Italy). Tukey’s HSD test was employed for correlating interaction means at 5% (of P < 0.05) probability level.

### Ethics approval and consent to participate

In this study, experimental research, and field studies on plants (either cultivated or wild), including the collection of plant material involved from University of Agriculture, Multan, Pakistan. All the protocols and experiment were conducted according to national, and international guidelines and legislation.

## Results

Statistical analysis elaborating significant (p ≤ 0.05) differences for salinity treatments, rootstocks (indigenous cucurbit landraces) and their interaction for cucumber crops’ shoot fresh and dry weights, root fresh and dry weights, root and shoot lengths, chlorophyll a and b contents, water use efficiency (WUE), stomatal conductance and sub-stomatal CO_2_, total phenolic contents (TPC), superoxide dismutase (SOD), peroxidase (POD), sodium (Na^+^), calcium (Ca^2+^) and potassium (K^+^) activities and fruit yield plant^–1^. The results elaborated that studied grafted cucumber plants onto different cucurbit landraces responded differently under different saline treatments {control (2.4 dSm^–1^) to 8 dSm^–1^}.

### Morphological attributes

Increasing salinity concentrations (2.4 to 8 dSm^–1^) significantly (P < 0.05) reduced the morphological parameters (SL, RL, SFW, RFW, SDW, RDW, SG, RG and NL) in non-grafted and grafted cucumber plants (Table 1). The cucumber plants grafted onto bottle gourd exhibited maximum shoot length (SL; 32.1%), shoot fresh weight (SFW; 33.3%), shoot dry weight (SDW; 39.3%), rootstock girth (RG; 12.21%), scion girth (SG; 14.5%), number of leaves (NL; 18%), root length (RL; 34%), root fresh weight (RFW; 12.2%) and root dry weight (RDW; 21%) compared to non-grafted plants under control growing conditions (2.4 dSm^–1^). Similarly, under saline environment (4 dSm^–1^), the highest SL (30.4%), SFW (35.7%), SDW (39%), RG (12.5%), SG (10%), NL (18.8%), RL (34%), RFW (20.3%) and RDW (31.3%) were recorded in cucumber plants grafted onto bottle gourd compared to non-grafted ones (Table 1). However, inter-nodal distance (ID) of non-grafted plants was 7% higher than that of cucumber grafted on bottle gourd under control (2.4 dSm^–1^) which started increasing with increasing saline (4 to 8 dSm^–1^) growing conditions (Table 1).

**Table 1:** Shoot length (SL) root length (RL), shoot fresh weight (SFW), root fresh weight (RFW), shoot dry weight (SDW), root dry weight (RDW), scion girth (SG), rootstocks girth (RG), number of leaves (NL) and inter-nodal distance (ID) of cucumber plants grafted on indigenous cucurbit landraces under saline conditions.

Rootstocks	Treatments (dS m^–1^)	SL (cm)	RL (cm)	SFW (g)	RFW (g)	SDW (g)	RDW (g)	SG (mm)	RG (mm)	NL	ID (mm)
Non-grafted cucumber F1	Control (2.4)	91.8 ± 2.5^f^	15.8 ± 0.03^f^	59.4 ± 0.4^e^	6.3 ± 0.02^fg^	18.2 ± 0.08^g^	3.4 ± 0.02^c^	2.90 ± 0.05^cd^	–	11.03 ± 0.03^e^	7.6 ± 0.2^eh^
	4	86.5 ± 1.1^fg^	14.4 ± 0.04^g^	56.3 ± 0.4^ef^	5.6 ± 0.04^i^	17.2 ± 0.09^gh^	2.6 ± 0.04^f^	2.6 ± 0.02^fg^	–	10.21 ± 0.04fg	7.5 ± 0.04^f-h^
	6	82.5 ± 1.4^gh^	13.2 ± 0.02^h^	53.5 ± 0.3^fg^	5.3 ± 0.04^jk^	15.4 ± 0.02^ij^	2.3 ± 0.04^g-i^	2.4 ± 0.04^jk^	–	9.6 ± 0.05^ij^	7.7 ± 0.03^d-h^
	8	77.8 ± 0.98^hi^	12.3 ± 0.04^j^	45.3 ± 0.2^hi^	5.1 ± 0.04^lm^	13.7 ± 0.2^m^	2.1 ± 0.05^h-l^	2.2 ± 0.01^k^	–	8.83 ± 0.05^k^	7.9 ± 0.04^b-f^
Bottle gourd + cucumber	Control (2.4)	135.4 ± 2.0^a^	24.0 ± 0.02^a^	89.0 ± 0.5^a^	7.2 ± 0.02^a^	30.0 ± 0.07^a^	4.2 ± 0.02^a^	3.3 ± 0.03^a^	3.11 ± 0.03^a^	13.44 ± 0.04^a^	6.9 ± 0.03^i^
	4	124.4 ± 1.6^bc^	22.0 ± 0.03^b^	87.7 ± 0.8^a^	7.1 ± 0.05^ab^	28.2 ± 0.02^b^	3.8 ± 0.04^b^	3.0 ± 0.02^b^	2.8 ± 0.01^b^	12.6 ± 0.05^b^	7.2 ± 0.04^hi^
	6	122.6 ± 1.4^b-d^	20.2 ± 0.04^c^	80.9 ± 0.5^b^	6.9 ± 0.02^cd^	25.4 ± 0.7^cd^	2.8 ± 0.02^ef^	2.9 ± 0.03^b^	2.7 ± 0.03^bc^	11.7 ± 0.06^d^	7.3 ± 0.02^g-i^
	8	115.9 ± 3.2^de^	19.0 ± 0.03^d^	77.8 ± 0.9^bc^	6.6 ± 0.05^de^	23.9 ± 0.04^e^	2.5 ± 0.02^fg^	2.5 ± 0.02^fg^	2.4 ± 0.02^ef^	10.2 ± 0.05^fg^	7.6 ± 0.04^d-h^
Sponge gourd + cucumber	Control (2.4)	74.9 ± 1.6^h-j^	13.1 ± 0.04^h^	48.2 ± 0.2^h^	6.0 ± 0.02^h^	14.8 ± 0.04^j-l^	2.0 ± 0.02^kl^	2.6 ± 0.03^ef^	2.6 ± 0.02^cd^	9.92 ± 0.02^j^	8.0 ± 0.03^b-d^
	4	70.6 ± 0.4^ij^	12.9 ± 0.02^i^	45.9 ± 0.2^hi^	5.3 ± 0.02^jk^	14.0 ± 0.05^lm^	2.3 ± 0.02^h-j^	2.5 ± 0.02^gh^	2.4 ± 0.02^ef^	9.8 ± 0.04^hi^	8.2 ± 0.9^a-c^
	6	67.8 ± 1.0^j^	11.7 ± 0.02^k^	41.7 ± 0.07^j^	5.1 ± 0.04^lm^	12.5 ± 0.04^n^	2.1 ± 0.04^h-l^	2.3 ± 0.04^jk^	2.2 ± 0.03^hi^	9.32 ± 0.04^j^	8.33 ± 0.11^a-c^
	8	65.7 ± 0.8^j^	10.0 ± 0.03^l^	39.9 ± 0.6^j^	4.9 ± 0.02^m^	11.7 ± 0.02^n^	1.9 ± 0.02^l^	1.9 ± 0.004^l^	1.9 ± 0.03^j^	8.22 ± 0.04^l^	8.5 ± 0.12^a^
Ridge gourd + cucumber	Control (2.4)	78.1 ± 0.6^hi^	14.4 ± 0.04^g^	56.5 ± 0.04^ef^	6.1 ± 0.09^gh^	16.3 ± 0.04^hi^	2.1 ± 0.09^j-l^	2.8 ± 0.02^cd^	2.6 ± 0.02^cd^	10.1 ± 0.05^fg^	7.8 ± 0.06^b-g^
	4	75.4 ± 0.6^h-j^	13.1 ± 0.04^h^	52.5 ± 0.4^g^	5.5 ± 0.01^ij^	15.1 ± 0.06^jk^	2.4 ± 0.02^gh^	2.7 ± 0.04^de^	2.6 ± 0.03^cd^	9.8 ± 0.02^hi^	8.0 ± 0.02^b-e^
	6	70.8 ± 0.4^ij^	12.3 ± 0.04^j^	46.6 ± 0.7^hi^	5.4 ± 0.04^j-l^	14.2 ± 0.09^k-m^	2.2 ± 0.04^h-k^	2.4 ± 0.02^h-j^	2.3 ± 0.04^gh^	9.4 ± 0.04^j^	8.1 ± 0.07^b-d^
	8	68.1 ± 0.5^j^	10.2 ± 0.02^l^	43.8 ± 0.9^ij^	5.0 ± 0.02^m^	13.8 ± 0.04^lm^	2.2 ± 0.02^h-k^	2.3 ± 0.02^jk^	1.9 ± 0.02^j^	8.6 ± 0.05^k^	8.2 ± 0.03^a-c^
Pumpkin + cucumber	Control (2.4)	130.4 ± 1.0^ab^	22.0 ± 0.02^b^	86.4 ± 0.6^a^	6.8 ± 0.04^bc^	29.0 ± 0.07^b^	3.7 ± 0.04^b^	3.22 ± 0.02^b^	2.9 ± 0.02^b^	12.5 ± 0.03^b^	7.2 ± 0.20^hi^
	4	122.7 ± 0.6^cd^	20.1 ± 0.05^c^	78.4 ± 0.6^bc^	6.4 ± 0.02^ef^	25.8 ± 0.07^c^	3.4 ± 0.02^c^	3.1 ± 0.01^de^	2.7 ± 0.04^bc^	12.2 ± 0.04^c^	7.4 ± 0.03^gh^
	6	117.7 ± 1.0^c-e^	19.0 ± 0.03^d^	75.4 ± 0.7^cd^	6.2 ± 0.02^f-h^	24.7 ± 0.06^de^	3.2 ± 0.02^d^	2.9 ± 0.03^g-i^	2.5 ± 0.03^de^	10.3 ± 0.05^f^	7.5 ± 0.03^g-h^
	8	111.9 ± 0.9^e^	18.7 ± 0.04^e^	72.6 ± 0.4^d^	6.0 ± 0.07^h^	22.0 ± 0.05^f^	2.9 ± 0.04^e^	2.4 ± 0.02^h-j^	2.3 ± 0.01^gh^	9.8 ± 0.04^hi^	7.7 ± 0.4^d-h^
HSD (Tukey) value	8.13	0.188	3.696	0.224	1.03	0.222	0.143	0.184	0.296	0.511	0.512
Rootstocks means	Non-graft cucumber f1	84.72 ± 1.3^b^	13.9 ± 0.04^c^	54.15 ± 0.35^c^	5.62 ± 0.04^c^	16.15 ± 0.13^c^	2.62 ± 0.04^b^	2.6 ± 0.02^c^	–	9.92 ± 0.04^c^	7.67 ± 0.079^c^
	Bottle gourd + cucumber	124.6 ± 1.1^a^	21.33 ± 0.0^a^	83.92 ± 0.74^a^	6.96 ± 0.03^a^	26.92 ± 0.17^a^	3.38 ± 0.03^a^	2.99 ± 0.03^a^	2.79 ± 0.029^a^	11.95 ± 0.05^a^	7.30 ± 0.032^d^
	Sponge gourd + cucumber	69.88 ± 1.3^c^	11.9 ± 0.03^e^	43.97 ± 0.30^e^	5.37 ± 0.03^e^	13.11 ± 0.04^e^	2.10 ± 0.03^d^	2.37 ± 0.024^d^	2.28 ± 0.031^e^	9.30 ± 0.04^d^	8.26 ± 0.091^a^
	Ridge gourd + cucumber	73.15 ± 1.31^c^	12.53 ± 0.04^d^	49.89 ± 0.61^d^	5.48 ± 0.05^d^	14.90 ± 0.06^d^	2.21 ± 0.04^c^	2.46 ± 0.03^c^	2.38 ± 0.31^d^	9.46 ± 0.041^c^	8.05 ± 0.05^b^
	Pumpkin + cucumber	120.5 ± 1.26^a^	19.97 ± 0.04^b^	78.27 ± 0.60^b^	6.42 ± 0.04^b^	25.36 ± 0.06^b^	3.37 ± 0.03^a^	2.90 ± 0.024^b^	2.59 ± 0.026^b^	11.20 ± 0.039^b^	7.46 ± 0.13^d^
HSD (Tukey) value	3.09	0.071	1.404	0.085	0.394	0.084	0.054	0.070	0.112	0.193	0.194
Treatments means	Control (2.4)	102 ± 1.6^a^	17.15 ± 0.54^a^	67.93 ± 0.46^a^	6.56 ± 0.04^a^	21.65 ± 0.06^a^	3.12 ± 0.04^a^	2.89 ± 0.03^a^	2.80 ± 0.030^a^	11.39 ± 0.034^a^	7.50 ± 0.105^c^
	4dSm^–1^	96 ± 0.84^b^	15.11 ± 0.4^b^	64.21 ± 0.49^b^	6.02 ± 0.03^b^	20.10 ± 0.09^b^	2.95 ± 0.034^b^	2.69 ± 0.025^b^	2.62 ± 0.027^b^	10.91 ± 0.042^b^	7.68 ± 0.045^b^
	6dSm^–1^	92 ± 1.1^c^	13.7 ± 0.5^c^	59.7 ± 0.49^c^	5.75 ± 0.035^c^	18.47 ± 0.14^c^	2.54 ± 0.037^c^	2.49 ± 0.033^c^	2.42 ± 0.037^c^	10.06 ± 0.050^c^	7.80 ± 0.054^a^
	8dSm^–1^	87 ± 1.4^d^	12.4 ± 0.4^d^	56.32 ± 0.63^d^	5.56 ± 0.04^d^	16.92 ± 0.07^d^	2.33 ± 0.035^d^	2.22 ± 0.01^d^	2.15 ± 0.021^d^	9.11 ± 0.048^d^	8.01 ± 0.103^a^
HSD (Tukey) value	2.66	0.060	1.180	0.071	0.331	0.070	0.045	0.058	0.094	0.163	0.163

Each value in table is a mean of 4 replicates. HSD (Tuckey Test) for grafting x treatment was significant at p ≤ 0.05 ± S.E. Means sharing different letters are significantly different at p ≤ 0.05.

### Gaseous exchange attributes

**Figure 1:**
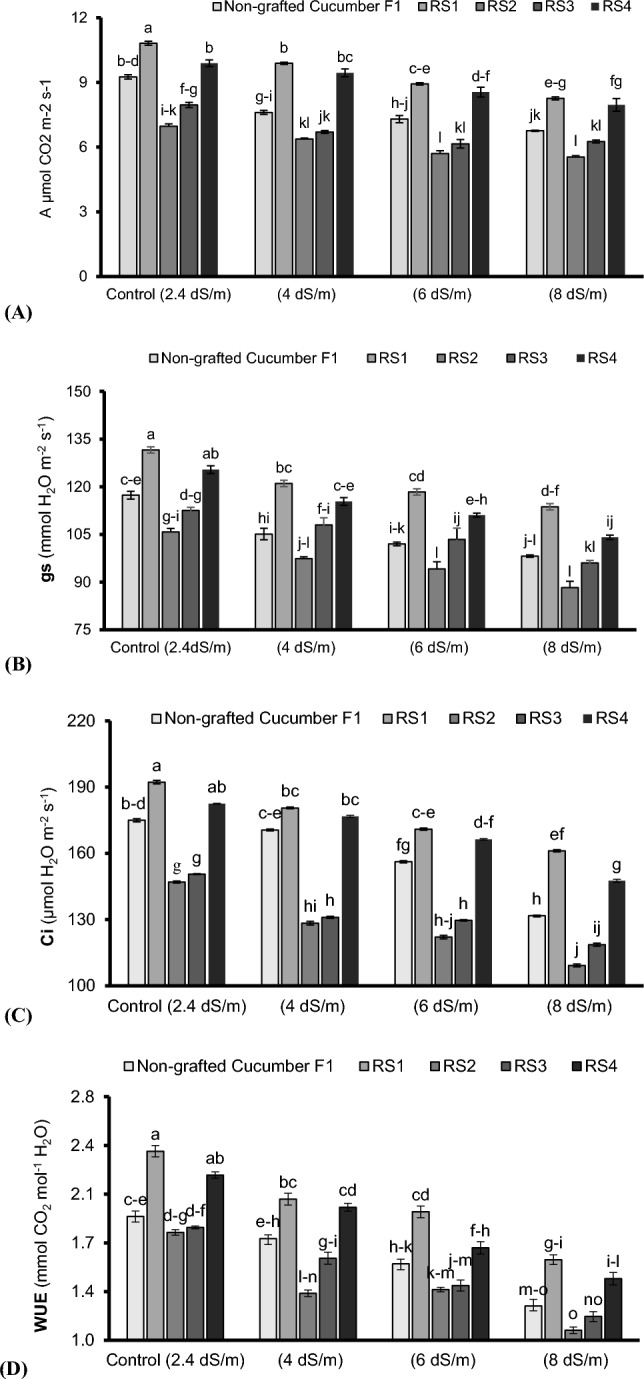
Photosynthetic Rate (*A*) (**A**), Stomatal Conductance (*gs*) (**B**), Sub-Stomatal CO_2_ (*Ci*) (**C**) and Water Use Efficiency (WUE) (**D**) of cucumber plants grafted on different cucurbits grown under various saline conditions. *RS1 (bottle gourd + Cucumber), RS2 (Sponge gourd + Cucumber) RS3 (Ridge Gourd + Cucumber) and RS4 (Pumpkin + Cucumber). Each value in figure is a mean of 4 replicates. HSD (Tuckey Test) for grafting x treatment was significant at p ≤ 0.05 ± S.E. Means sharing different letters are significantly different at p ≤ 0.05.

Increasing salinity levels (2.4 to 8 dSm^–1^) significantly (P < 0.05) reduced the photosynthetic rate (*A*), stomatal conductance (*gs*), sub-stomatal CO_2_ (*Ci*), and water use efficiency (WUE) in non-grafted and grafted cucumber plants (Fig. 1A–D). However, the cucumber plants grafted onto bottle gourd attained the highest *A* (1.15 fold), *gs* (1.09 fold), *Ci* (1.12 fold), and WUE (1.24 fold) relative to non-grafted ones under control growing conditions (2.4 dSm^-1^). About 1.15-fold, 1.09-fold, 1.12-fold, and 1.24-fold increase were noted in *A*, *gs*, *Ci*, and WUE, respectively, in cucumber plants grafted on bottle gourd compared to non-grafted ones under control (2.4 dSm-1). Among saline growing environment (4–8 dSm^–1^), maximum *A* (1.26-fold), *gs* (1.05-fold), *Ci* (1.15-fold), and WUE (1.16-fold) were noted in cucumber plants grafted onto bottle gourd relative to non-grafted ones under 4 dSm^-1^ (Fig. 1A–D).

### Biochemical attributes

**Figure 2:**
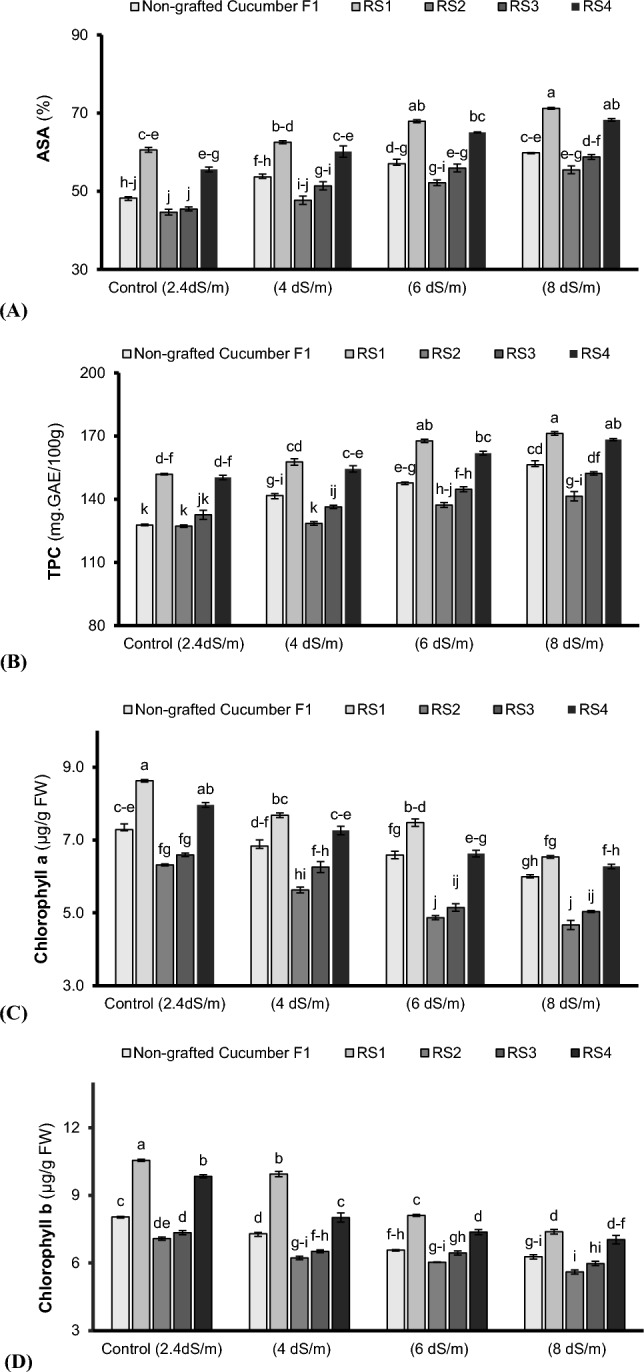
Antioxidants scavenging activity (ASA) (**A**), total phenolics contents (TPC) (**B**), chlorophyll a (**C**) and chlorophyll b (**D**) of cucumber plants grafted on different cucurbits grown under various saline conditions. *RS1 (bottle gourd + Cucumber), RS2 (Sponge gourd + Cucumber) RS3 (Ridge Gourd + Cucumber) and RS4 (Pumpkin + Cucumber). Each value in figure is a mean of 4 replicates. HSD (Tuckey Test) for grafting x treatment was significant at p ≤ 0.05 ± S.E. Means sharing different letters are significantly different at p ≤ 0.05.

**Figure 3:**
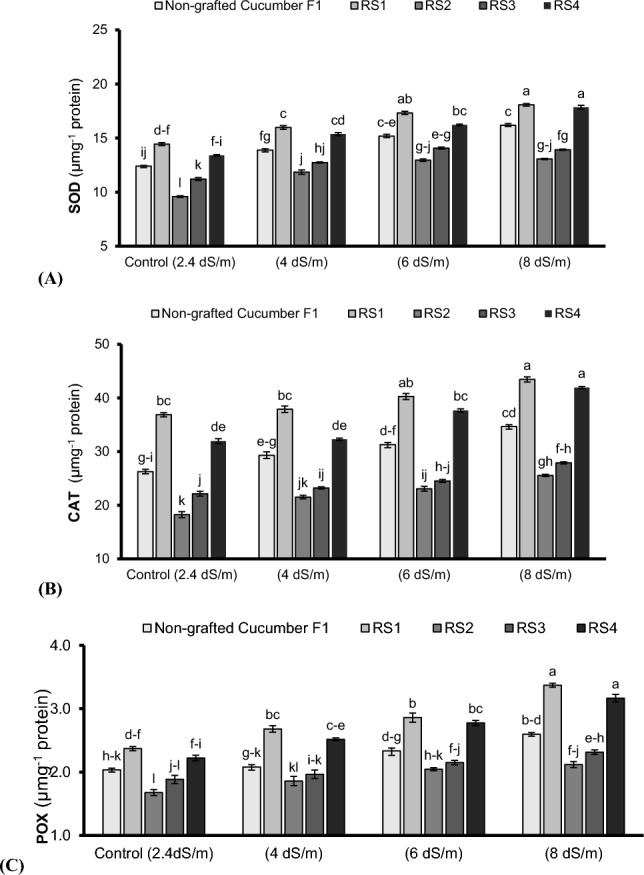
Superoxidase dismutase (SOD) (**A**), Catalase (CAT) (**B**) and peroxidase (POX) (**C**) of cucumber plants grafted on different cucurbits grown under various saline conditions. *RS1 (bottle gourd + Cucumber), RS2 (Sponge gourd + Cucumber) RS3 (Ridge Gourd + Cucumber) and RS4 (Pumpkin + Cucumber). Each value in figure is a mean of 4 replicates. HSD (Tuckey Test) for grafting x treatment was significant at p ≤ 0.05 ± S.E. Means sharing different letters are significantly different at p ≤ 0.05.

Total antioxidant scavenging activity (ASA) (Fig. 2A), total phenolic contents (TPC) (Fig. 2B), superoxide dismutase (SOD) (Fig. 3A), catalase (CAT) (Fig. 3B), and peroxidase (POX) activities (Fig. 3C) were enhanced significantly (P < 0.05) with an increase in salinity stress (2.4–8 dSm^–1^) in non-grafted and grafted cucumber plants. Maximum ASA (Fig. 2A), TPC (Fig. 2B), SOD (Fig. 3A), CAT (Fig. 3B), and POX (Fig. 3C) were noted in cucumber plants grafted on indigenous bottle gourd land race under the highest saline growing conditions (8 dSm^–1^). The cucumber plants grafted on bottle gourd exhibited 16%, 8.7% 10.4%, 20.2%, and 22.9% enhancement in ASA, TPC, SOD, CAT, and POX respectively, compared to non-grafted ones under 8 dSm^-1^. However, increasing salinity significantly (P < 0.05) reduced the chlorophyll-a and -b contents. Maximum chlorophyll-a (23.8%) and chlorophyll-b (15.56%) were noted in bottle gourd grafted cucumber plants compared to non-grafted ones under control (2.4 dSm^–1^). However, under saline growing conditions (4–8 dSm^–1^), the highest chlorophyll-a (26.6%) (Fig. 2C) and chlorophyll-b (11%) (Fig. 2D) contents were observed in bottle gourd grafted cucumber plants grown in 4 dSm^–1^ salinity level, than that of non-grafted ones.

### Ionic attributes

Increase in saline stress (2.4–8 dSm^–1^) significantly (P < 0.05) but negatively affected K^+^ and Ca^2+^ contents while positively enhanced the Na^+^ contents in leaves and roots of non-grafted and grafted cucumber plants (Table 2). The highest K^+^ and Ca^2+^ uptake was detected in cucumber plants grafted on bottle gourd under control growing conditions (Table 2). The cucumber plants grafted on bottle gourd exhibited higher uptake of K^+^ (1.10-fold in leaves and 1.18-fold in roots) and Ca^2+^ (1.26-fold and 1.47-fold) than non-grafted ones under control (2.4 dSm^–1^). Similar trend observed under saline growing conditions (4–8 dSm^–1^) where maximum K^+^ uptake (1.16-fold in leaves and 1.24-fold in roots) and Ca^2+^ uptake (1.24-fold in leaves and 1.77-fold in roots) were recorded under 4 dSm^–1^ in cucumber plants grafted onto bottle gourd compared to non-grafted ones (Table 2). However, minimum Na^+^ uptake (0.86-fold in leaves and 0.71-fold in roots) was noted in cucumber plant grafted onto bottle gourd under control (2.4 dSm^–1^). However, maximum Na^+^ uptake was recorded in cucumber plants grafted onto sponge gourd at a salinity level of 8 dSm^–1^ (Table 2).

**Table 2:** Sodium (Na^+^), Potassium (K^+^), and Calcium (Ca^2+^) in roots and shoots of cucumber plants grafted on different cucurbits grown under various saline conditions.

Rootstocks	Treatments (dS m^–1^)	Na^+^ in roots (mg.Kg^–1^)	K^+^ in roots (mg.Kg^–1^)	Ca^2+^ in roots (mg.Kg^–1^)	Na^+^ in leaves (mg.Kg^–1^)	K^+^ in leaves (mg.Kg^–1^)	Ca^2+^ in leaves (mg.Kg^-1^)
Non-grafted cucumber F1	Control (2.4)	1.7 ± 0.04^i^	29.3 ± 0.53^c^	13.81 ± 0.13^e^	2.66 ± 0.043^jk^	39.55 ± 0.53^c^	29.81 ± 0.65^f^
	4	4.9 ± 0.05^f^	24.26 ± 0.46^f-h^	10.32 ± 0.12^f^	5.87 ± 0.046^g^	34.55 ± 0.55^fg^	27.92 ± 0.36^g^
	6	6.12 ± 0.043^d^	20.11 ± 0.34^jk^	8.80 ± 0.07^g^	7.11 ± 0.041^d^	30.22 ± 0.34^ij^	26.14 ± 0.32^h^
	8	8.0 ± 0.023^ab^	18.44 ± 0.51^kl^	8.07 ± 0.05^gh^	8.55 ± 0.022^b^	27.85 ± 0.87^kl^	23.07 ± 0.05^j^
Bottle gourd + cucumber	Control (2.4)	1.18 ± 0.063^k^	34.88 ± 0.25^a^	20.33 ± 0.23^a^	2.29 ± 0.06^k^	43.40 ± 0.21^a^	37.85 ± 0.10^a^
	4	3.3 ± 0.05^g^	30.29 ± 0.32^bc^	18.31 ± 0.11^b^	4.29 ± 0.05^i^	40.25 ± 0.31^bc^	34.80 ± 0.30^b^
	6	5.3 ± 0.03^ef^	27.18 ± 0.42^de^	16.77 ± 0.15^c^	6.25 ± 0.03^e-g^	37.25 ± 0.52^de^	32.88 ± 0.16^cd^
	8	6.1 ± 0.05^d^	23.33 ± 0.44^g-i^	15.07 ± 0.05^de^	7.11 ± 0.045^d^	33.33 ± 0.44^gh^	30.47 ± 0.20^ef^
Sponge gourd + cucumber	Control (2.4)	1.98 ± 0.16^h^	25.25 ± 0.44^e-g^	20.25 ± 0.31^a^	3.0 ± 0.16^j^	35.40 ± 0.32^f-g^	24.84 ± 0.10^hi^
	4	5.58 ± 0.12^e^	21.81 ± 0.17^ij^	8.66 ± 0.23^gh^	6.58 ± 0.13^e^	32.55 ± 0.22^hi^	23.66 ± 0.23^ij^
	6	7.65 ± 0.19^ab^	19.33 ± 0.32^kl^	7.47 ± 0.23^h^	8.66 ± 0.19^ab^	29.33 ± 0.32^jk^	22.47 ± 0.23^jk^
	8	8.3 ± 0.092^a^	15.33 ± 0.44^m^	5.03 ± 0.02^i^	9.29 ± 0.09^a^	25.51 ± 0.36^l^	20.03 ± 0.02^k^
Ridge gourd + cucumber	Control (2.4)	1.80 ± 0.043^i^	25.81 ± 0.30^ef^	10.99 ± 0.40^f^	2.77 ± 0.041^jk^	36.14 ± 0.50^ef^	25.99 ± 0.40^h^
	4	5.22 ± 0.09^ef^	23.44 ± 0.31^g-i^	10.21 ± 0.07^f^	6.03 ± 0.07^fg^	33.44 ± 0.31^gh^	25.21 ± 0.07^hi^
	6	6.66 ± 0.01^c^	20.22 ± 0.29^jk^	8.73 ± 0.26^gh^	7.66 ± 0.01^c^	30.18 ± 0.31^ij^	23.73 ± 0.26^ij^
	8	7.87 ± 0.05^ab^	17.47 ± 0.20^lm^	6.03 ± 0.02^i^	8.87 ± 0.05^ab^	27.47 ± 0.20^kl^	21.03 ± 0.02^k^
Pumpkin + cucumber	Control (2.4)	1.33 ± 0.01^jk^	32.55 ± 0.65^b^	19.03 ± 0.02^ab^	2.33 ± 0.04^k^	41.81 ± 0.28^ab^	34.29 ± 0.66^bc^
	4	4.21 ± 0.09^g^	28.62 ± 0.28^cd^	16.32 ± 0.23^cd^	5.21 ± 0.09^h^	39.03 ± 0.21^cd^	33.31 ± 0.11^b-d^
	6	5.40 ± 0.07^e^	26.17 ± 0.25^ef^	15.07 ± 0.05^de^	6.4 ± 0.069^ef^	35.47 ± 0.20^e-g^	31.77 ± 0.15^de^
	8	6.55 ± 0.045^cd^	22.3 ± 0.56^h-j^	14.10 ± 0.12^e^	7.54 ± 0.04^cd^	32.03 ± 0.56^hi^	30.07 ± 0.05^ef^
HSD (Tukey) value		0.488	2.281	1.303	0.466	0.110	0.757
Rootstocks means	Non-graft cucumber F1	5.041 ± 0.04^c^	23.09 ± 0.46^c^	10.25 ± 0.09^c^	6.04 ± 0.038^c^	33.04 ± 0.57^c^	26.73 ± 0.34^c^
	Bottle gourd + Cucumber	3.97 ± 0.05^e^	28.92 ± 0.36^a^	17.62 ± 0.14^a^	4.99 ± 0.045^e^	38.56 ± 0.37^a^	34.00 ± 0.19^a^
	Sponge gourd + cucumber	6.00 ± 0.15^a^	20.43 ± 0.34^e^	7.85 ± 0.20^e^	6.88 ± 0.146^a^	30.70 ± 0.31^e^	22.75 ± 0.14^e^
	Ridge gourd + cucumber	5.4 ± 0.051^b^	21.73 ± 0.28^d^	8.99 ± 0.19^d^	6.33 ± 0.041^b^	31.81 ± 0.33^d^	23.99 ± 0.19^c^
	Pumpkin + cucumber	4.4 ± 0.053^d^	27.34 ± 0.44^b^	16.13 ± 0.10^b^	5.37 ± 0.052^d^	37.08 ± 031^b^	32.36 ± 0.24^b^
HSD (Tukey) value		0.185	0.855	0.495	0.177	0.155	0.667
Treatments means	Non-saline	1.59 ± 0.064^a^	29.61 ± 0.43^a^	14.89 ± 0.22^a^	2.61 ± 0.06^a^	39.26 ± 0.37^a^	30.56 ± 0.38^a^
	4dSm^–1^	4.64 ± 0.083^b^	26.69 ± 0.31^b^	12.77 ± 0.15^b^	5.60 ± 0.079^b^	35.97 ± 032^b^	28.98 ± 0.21^b^
	6dSm^–1^	6.23 ± 0.071^c^	22.60 ± 0.33^c^	11.37 ± 0.15^c^	7.22 ± 0.067^c^	32.49 ± 0.34^c^	27.40 ± 0.22^c^
	8dSm^–1^	7.20 ± 0.050^d^	19.32 ± 0.43^d^	9.66 ± 0.05^d^	8.27 ± 0.050^d^	29.24 ± 0.49^d^	24.94 ± 0.07^d^
HSD (Tukey) value		0.155	0.728	0.416	0.149	0.130	0.560

Each value in table is a mean of 4 replicates. HSD (Tuckey Test) for grafting x treatment was significant at p ≤ 0.05 ± S.E. Means sharing different letters are significantly different at p ≤ 0.05.

### Yield related attributes

**Figure 4:**
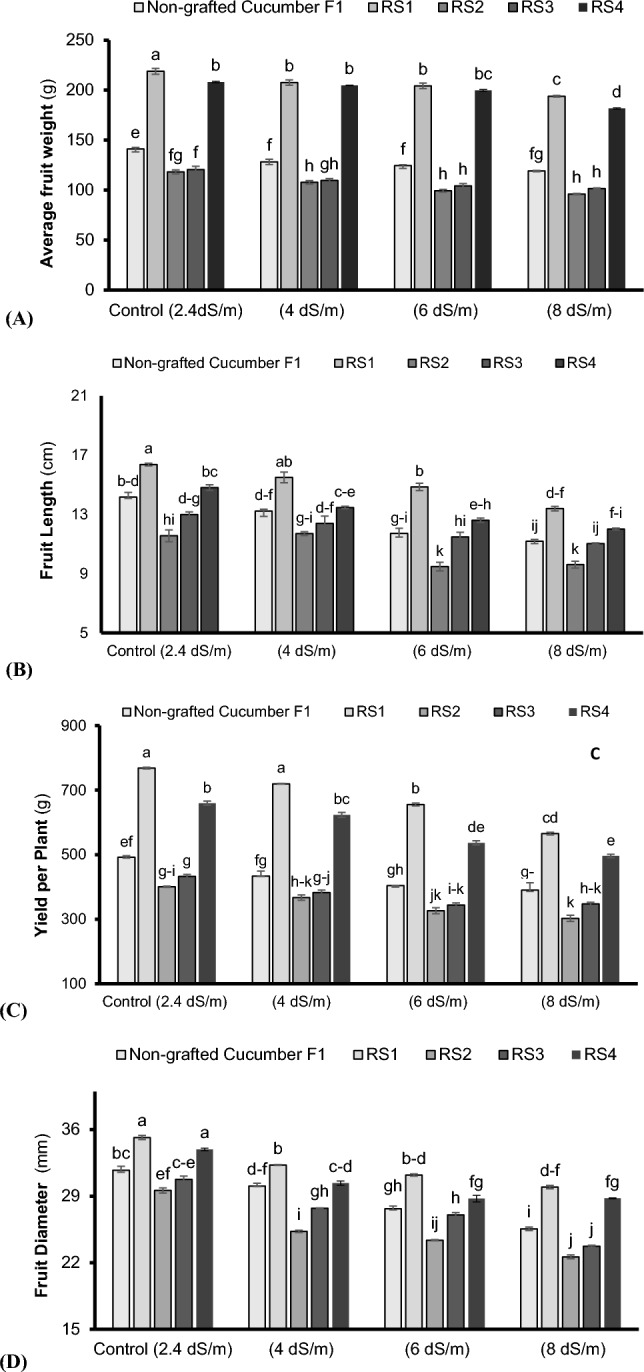
Average fruit weight (**A**), fruit length (**B**), yield per plant (**C**) and fruit diameter (**D**) of cucumber plants grafted on different cucurbits grown under various saline conditions. *RS1 (bottle gourd + Cucumber), RS2 (Sponge gourd + Cucumber) RS3 (Ridge Gourd + Cucumber) and RS4 (Pumpkin + Cucumber). Each value in figure is a mean of 3 replicates. HSD (Tuckey Test) for grafting x treatment was significant at p ≤ 0.05 ± S.E. Means sharing different letters are significantly different at p ≤ 0.05.

Saline growing conditions (4 to 8 dSm^–1^) negatively affected the average fruit weight (Fig. 4A), length (Fig. 4B), diameter (Fig. 4C), and yield per plant (Fig. 4D) in non-grafted and grafted cucumber plants. However, maximum yield related attributes were noted in cucumber plants grafted onto bottle gourd followed by pumpkin under control (2.4 dSm^–1^) (Fig. 4A–D). The cucumber plants grafted on bottle gourd exhibited 35.2%, 13.4%, 35.9% and 9.8% improvement in average fruit weight (Fig. 4A), length (Fig. 4B), diameter (Fig. 4C), and yield per plant (Fig. 4A), respectively, compared to non-grafted ones under control growing environment (2.4 dSm^–1^). Similar trend observed under saline growing conditions, where maximum increase was observed under 4 dSm^–1^ i.e. 38.2%, 14.7%, 39.7% and 6.9% in average fruit weight (Fig. 4A), length (Fig. 4B), diameter (Fig. 4C), and yield per plant (Fig. 4D) respectively, compared to non-grafted ones.

### Principal component analysis (PCA)

**Figure 5:**
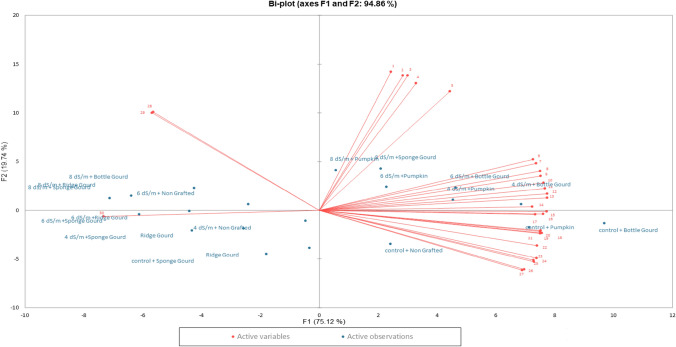
Linkage map of all variables and factors by using principal component analysis. The parameters are coded as 1. Superoxide dismutase, 2. Peroxidase, 3. Total phenolics contents, 4. Antioxidants scavenging activity, 5. Catalase, 6. Average fruit weight, 7. Shoot length, 8. Shoot fresh weight, 9. Shoot dry weight, 10. Root length, 11. Yield per plant, 12. Calcium in leaves, 13. Calcium in roots, 14. Root dry weight, 15. Photosynthetic rate, 16. Sub-stomatal conductance, 17. Root fresh weight, 18. Chlorophyll a, 19. Chlorophyll b, 20. Number of leaves, 21. Stomatal conductance, 22. Fruit diameter, 23. Potassium in roots, 24. Potassium in leaves, 25. Water use efficiency, 26. Rootstocks girth, 27. Scion girth, 28. Sodium in leaves, 29. Sodium in roots and 30. Internodal distance.

A linkage map was created using principal component analysis (PCA) of all variables and factors (Fig. 5) based on first two components that accounted for approximately 94.7% of the overall variation. The bi-plot analysis elaborates that under non-stressed growing conditions, indigenous bottle gourd landrace exhibited maximum performance in enhancing the studied parameters and showed a strong grouping with photosynthetic pigments (Chl. a & b), number of leaves, and stomatal conductance. Further, the PCA revealed that under saline conditions (4 dSm^–1^), indigenous bottle gourd landrace performed well in improving the studied parameters such as shoot length, shoot fresh and dry weight, calcium uptake in leaves and roots, and average fruit weight, thereby, yield. In the same way, the bottle gourd landrace showed higher performance at a higher salinity level (6 dSm^–1^) as well, hence, helped in improving the studied morpho-physio-biochemical and yield-related attributes. Besides, the indigenous bottle gourd landrace at 4 dSm^–1^ exhibited a strong negative correlation with sodium uptake both in roots and leaves. This reduced uptake of sodium ions is the main reason for increased salt tolerance potential of bottle gourd indigenous landrace (Fig. 5).

## Discussion

Salt stress disturbs natural behavior of the agricultural crops including vegetables^33^. The presence of salts particularly NaCl in the rhizosphere adversely affects plant growth and productivity^34,35^. In this study, an increase in salt concentration (4 to 8 dSm^–1^) negatively affected the morphological (shoot length, root length, shoot fresh weight, root fresh weight and the number of leaves) attributes except inter-nodal distance which increased with enhancing salinity (Table 1). This reduction in the morphological parameters may be attributed to loss of turgor and reduction in cellular expansion, thereby inhibiting the growth of tissues and organs^36,37^. In the current study, rootstock induced salt tolerance in grafted cucumber plants by improving the morpho-physio-biochemical, ionic and yielding traits. The plants grafted onto bottle gourd showed maximum performance for biomass attributes compared to others cucurbit landraces (Table 1) probably due to production of more cytokinin and efficient utilization of xylem sap to transport water and nutrients to the shoot system, hence, promoted the plant growth and productivity^38^. During current study, increasing salt concentrations (4 to 8 dSm^–1^) in root zone environment lead to denaturation of chlorophyll pigments (Fig. 2C,D) and disturbed gaseous exchange attributes (*A*, *gs*, *Ci* and WUE) (Fig. 1A–D) possibly by disrupting plant’s metabolic pathway^39^, deceasing β-carotene contents, distorting chloroplast and wrinkling of cell membrane^40^. However, gaseous exchange attributes were significantly improved in cucumber plants followed by pumpkin grafted onto bottle gourd relative to grafted and non-grafted plants under saline condition (Fig. 1A–D) perhaps due to the protective influence of grafting through up-regulating the intake/flow of CO_2_, promoting the Rubisco activity^38,41^ and generating differential microRNAs expression from the rootstock via phloem to the scion, hence, could be considered of high relevance to biological and metabolic processes^42^. Furthermore, during salt stress, bottle gourd might activate gene expression of the enzymes related to ribulose-1,5-bisphosphate (RuBP) regeneration that resulted in improved the photosystem efficiency^26^. Additionally, indigenous bottle gourd landrace as a rootstock induced the salt tolerance probably by an over expression of Arabidopsis H^+^ pyrophosphatase AVP1 genes, and earlier closure of stomata in grafted cucumber plants to sustain the hydration status, higher relative water contents and photosystem II quantum yield, regulate the plant growth, development and higher biomasses^43,44^. Moreover, the photosystem efficiency, ascorbic acid contents and sugar acid ratio enhancement is another possibility that embarks the salt tolerance induction^45,46^. Likewise, salt stress positively affects the plant’s anti-oxidative activities (Figs. 2, 3). In present study, the antioxidant scavenging activity (ASA) (Fig. 2A), total phenolic contents (TPC) (Fig. 2B) and antioxidant enzymes’ activities including SOD (Fig. 3A), POX (Fig. 3C), and CAT (Fig. 3B) predominately improved in cucumber plants grafted onto bottle gourd and pumpkin relative to non-grafted plants under saline condition. Our results are in agreement with Taïbi et al.^47^ and Elsheery et al.^48^, they reported higher ASA, CAT, POX, and SOD activities along-with rapid increase in H_2_O_2_ breakdown in plant cell of grafted plants under saline environments than non-grafted plants which confirmed dismutation potential of grafted plants^49^. Elevated undesirable inorganic ions like Na^+^ in rhizosphere solution disrupts K^+^ and Ca^2+^ acquisition by plant’s roots^50^ as observed during the current study where increasing salt levels (4 to 8 dSm^–1^) negatively affected the K^+^ and Ca^2+^ contents in roots and leaves (Table 2). This might be due to the competition of Na^+^ with K^+^ to enter the root. Na^+^ concentration increases in plants under saline condition^51^. Maintenance of high K^+^: Na^+^ is crucial for salt tolerance induction^52^. In the present study, higher K^+^ uptake was noted in the cucumber plants grafted onto bottle gourd followed by pumpkin landraces as compared to non-grafted ones (Table 2) possibly due to their potential to restricts Na^+^ in the root zone and improved K^+^ uptake, thereby, cellular homeostasis^25,46,53^. Recently, Zhang et al.^54^ and Wu et al.^55^ reported salt tolerance genes from high-quality genome sequences for luffa confirmed its salt tolerance induction mechanism. Pumpkin rootstock limits Na^+^ uptake by upregulating CmHKT1;1 (high affinity Na^+^ selective uniporter, preferably express in root stele and localized in plasma membrane). Overexpression of CmHKT1;1 limits the Na^+^ transport possibly either by its unloading from xylem transpiration stream or recirculation from shoot-to-root^19^ or increasing K^+^ accumulation^56^. Moreover, salt tolerance in pumpkin grafted cucumber plants is carried out probably by enhancing root Na^+^ exclusion through Na^+^/H^+^ antiporter triggered by the plasma membrane H^+^-ATPase and higher transcription for PMA and SOS1 (pre-requisite for sustaining cell K^+^/Na^+^ homeostasis)^43^. Calcium (Ca^2+^) uptake plays a central role in membrane integrity^57^. Salinity increases membrane susceptibility due to higher Na^+^ ions, reduction in Ca^+^ ions and generation of malondialdehyde (MDA) contents^47,58,59^. However, Ca^2+^ uptake is linked with the corresponding decrease in Na^+^ uptake during salt stress^60^.

It is observed that Higher calcium uptake reduces the threat of salinity via facilitating higher Ca^2+^: Na^+^/K^+^: Na^+^. During the current research work, an increase in Ca^2+^ uptake relative to Na^+^ in the cucumber plants grafted onto the indigenous bottle gourd landrace (Table 2) induced salinity tolerance probably due to higher Ca^2+^: Na^+^ selectivity^61^, reduction in Na^+^ influx via blocking non-selective cation channel (NSCC) and inhibiting K^+^ efflux through GORK channel, thereby, Ca^2+^ promotes membrane stability^62^. In addition, vacuolar and cytosolic Ca^2+^ block the fast vacuole (FV) channel in voltage voltage-dependent and independent way^63^ which stops the leaking of Na^+^ from the vacuole and their transportation into the cell^62^. In this way, calcium uptake promoted higher K^+^: Na^+^ under a saline environment (Table 2). This vigorous indigenous rootstock approach aids in capturing and transporting large amounts of ions to scion, higher concentrations of sugars, enzymes, and amino acids along-with secretion of organic acids which are vital for nutrient availability, mobilization, and their uptake in soil^64^. The adverse impact of rising salinity levels (4 to 8 dSm^–1^) on the cucumber yield could be related to higher salt concentration in roots and leaves as depicted in the current study (Fig. 4C). Yield traits were improved in grafted plants compared to non-grafted plants under saline growing medium (Fig. 4). However, the cucumber plants grafted onto the indigenous bottle gourd and pumpkin landraces exhibited higher yield and its related traits as compared to other indigenous ridge and sponge gourd landraces (Fig. 4A–D) possibly due to the ability of indigenous bottle gourd and pumpkin landraces to inhibits the accumulation or transfer of surplus Na^+^ ions (as depicted in present study) from either entry into the roots or being transported to the leaves^37,65^. Besides, an increase in gene expression and hormonal synthesis particularly isopenthyl adenosine transferase may be the reason that boosts up the cytokinin and trans-zeatin concentration, thereby, significant increase in yield traits^66^. The increased cytokinin concentrations delayed the stomatal closure and leaf senescence by increasing the plant leaf area and the K^+^ status together with reduction in concentration of toxic ions (e.g. Na^+)^ and hormones (e.g. abscisic acid). This enhancement in cytokinin concentration may also be the possible reason for the improvement in yield^66,67^.

## Conclusion

Salt stress imposed negative impacts on cucumber growth and productivity; however, grafting improved the salt tolerance potential in cucumber plants. The grafted cucumber onto indigenous bottle gourd landrace revealed higher growth, ionic contents, photosynthetic and water use efficiency as well as productivity under a moderate saline environment (4–6 dSm^–1^). The antioxidative activities were also found higher in cucumber plants grafted with indigenous bottle gourd used as rootstocks than pumpkin, sponge, and ridge gourd, subsequently helping the plants to evade salinity-induced effects. Further, field studies should be carried out to explore the potential of indigenous bottle gourd landrace as rootstock under field saline conditions for the possible development of hybrid rootstocks to arid and semi-arid climates.

## Data Availability

The datasets used and/or analyzed during the current study available from the corresponding author on reasonable request.
