# Author Correction: Cucumber grafting on indigenous cucurbit landraces confers salt tolerance and improves fruit yield by enhancing morpho-physio-biochemical and ionic attributes

**DOI:** 10.1038/s41598-024-53465-7

**Published:** 2024-02-05

**Authors:** Fazal Abbas, Hafiz Nazar Faried, Gulzar Akhtar, Sami Ullah, Talha Javed, Muhammad Asif Shehzad, Khurram Ziaf, Kashif Razzaq, Muhammad Amin, Fahad Masoud Wattoo, Aqsa Hafeez, Mehdi Rahimi, Amany H. A. Abeed

**Affiliations:** 1 Department of Horticulture, MNS University of Agriculture, Multan, Pakistan; 2 College of Agriculture, Fujian Agriculture and Forestry University, Fuzhou, 350002 China; 3 Institute of Plant Breeding and Biotechnology, MNS University of Agriculture, Multan, Pakistan; 4 Institute of Horticultural Sciences, University of Agriculture, Faisalabad, Pakistan; 5 Department of Horticultural Sciences, The Islamia University of Bahawalpur, Bahawalpur, Pakistan; 6 Department Plant Breeding and Genetics, PMAS Arid Agriculture University, Rawalpindi, Pakistan; 7 Department of Plant Sciences, Quaid-i-Azam University, Islamabad, 45320 Pakistan; 8 Department of Biotechnology, Institute of Science and High Technology and Environmental Sciences, Graduate University of Advanced Technology, Kerman, Iran; 9 Department of Botany and Microbiology, Faculty of Science, Assiut University, Assiut, 71516 Egypt

Correction to: *Scientific Reports* 10.1038/s41598-023-48947-z, published online 07 December 2023

The original version of this Article contained an error in Affiliation 3, which was incorrectly given as ‘Department of Agronomy, MNS University of Agriculture, Multan, Pakistan’. The correct affiliation is listed below.

Institute of Plant Breeding and Biotechnology, MNS University of Agriculture, Multan, Pakistan.

The original Article has been corrected.

